# Eco‐Friendly Solvent System for Inkjet Deposition of Wide Bandgap Perovskite Solar Cells Enabling Tandem Integration

**DOI:** 10.1002/advs.76463

**Published:** 2026-07-09

**Authors:** Uma Kousalya Dangudubiyyam, Raphael Pesch, Ozan Karakaya, Theresa Kuechle, Faranak Sadegh, Nils W. Rosemann, Henry Weber, Ralf Niemann, Fabian Fertig, Johannes Sutter, Jinzhao Li, Gerardo Hernandez Sosa, Ulrich W. Paetzold

**Affiliations:** ^1^ Light Technology Institute (LTI) Karlsruhe Institute of Technology Karlsruhe Germany; ^2^ InnovationLab Heidelberg Germany; ^3^ Institute of Microstructure Technology Karlsruhe Institute of Technology Eggenstein‐Leopoldshafen Germany; ^4^ Hanwha Qcells GmbH Bitterfeld‐Wolfen Germany; ^5^ Institute for Automation and Applied Informatics Karlsruhe Institute of Technology Eggenstein‐Leopoldshafen Germany

**Keywords:** green solvents, inkjet printing, perovskite solar cell, sustainable photovoltaics, tandem solar cell, γ‐valerolactone (GVL)

## Abstract

Inkjet printing offers scalable, material‐efficient route to fabricate perovskite solar cells. However, the widespread use of toxic and hazardous solvents in one‐step printed perovskite absorber layers poses significant environmental and safety challenges when handling large volumes in production. In this work, we overcome this limitation by demonstrating high‐performance, wide‐bandgap inkjet‐printed perovskite solar cells processed with truly green, non‐toxic solvent system, entirely free of carcinogenic and highly hazardous components. γ‐Valerolactone, a biomass‐derived solvent, is employed as the primary solvent due to its low toxicity and environmental impact, and dimethyl sulfoxide is introduced as a co‐solvent to overcome solubility constraints of wide‐bandgap perovskite precursors (> 1.68 eV). The ink chemistry and rheology are optimized for solubility and printability. The challenges of wetting and drying dynamics are addressed by tuning interactions between substrate and ink, combined with additive and surface engineering to enhance performance. The resulting green‐solvent‐based, inkjet‐printed perovskite solar cells exhibit power conversion efficiencies exceeding 17% in single‐junction devices. Critically, we further demonstrate first perovskite/silicon tandem solar cell integrating one‐step inkjet‐printed perovskite thin film, achieving an efficiency above 28%. These results establish green solvent inkjet printing as a viable and compelling pathway toward sustainable and scalable manufacturing of high‐efficiency perovskite photovoltaics.

## Graphical abstract

The fabrication of wide‐bandgap perovskite thin films via green solvents integrated with inkjet printing is developed. Controlled crystallization of the perovskite layer is achieved by optimizing ink formulation and additive engineering, enabling power conversion efficiencies above 17% for single junctions and 28% for perovskite/silicon tandem solar cells, demonstrating a scalable and sustainable pathway toward perovskite photovoltaics.

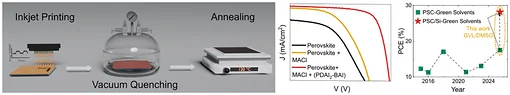

## 1 Introduction

Perovskite solar cells (PSCs) have emerged as a highly promising next‐generation photovoltaic (PV) technology with exceptional power conversion efficiencies (PCEs) exceeding 27% in under 15 years of development [1]. This remarkable progress is primarily attributed to the excellent optoelectronic characteristics of perovskite semiconductor thin films and their tunable bandgap (ranging from 1.2 to 2.3 eV) [2], which can be precisely engineered through compositional modifications. The bandgap tunability is a key factor in the promise of perovskite materials for tandem PV applications. Tandem solar cells (TSCs) combining perovskite and silicon semiconductors have achieved record‐breaking certified PCEs of up to 34.9% [3]. To transfer research to large‐scale manufacturing, additional challenges, such as long‐term stability and scalability, must be addressed [4, 5] Concerning scalability, a wide range of methods are explored in industry and academia, such as spray coating [6], inkjet printing [7, 8], evaporation [9, 10], blade coating [11], and slot‐die coating [12], to enable large‐area, cost‐effective perovskite production. Key performance metrics are the quality of the material, deposition throughput, production yield, and environmental hazards associated with the fabrication. Inkjet printing, a leading technology in the printed electronics industry, is known for its high precision, design flexibility, low material consumption, and cost‐effective operation [13, 14]. Since its initial application to perovskite deposition by Wei et al. in 2014 [14], it has gained substantial attention for fabricating active layers in PSCs. Notably, Eggers et al. achieved a PCE of 21.6% using inkjet‐printed perovskite films of 1 µm thickness [7]. More recently, advances in inkjet printing have also focused on controlling stoichiometry and interface properties, leading to further improvements in device performance and reproducibility [15, 16, 17]. Despite these promising results, industrial production of PSCs remains a challenge due to the use of N,N‐dimethylformamide (DMF), a solvent known to be toxic to both humans and the environment.

To address these concerns, recent research has shifted toward developing environmentally friendly solvents for perovskite thin‐film fabrication, aiming to reduce toxicity and enhance the sustainability of the process [18]. For instance, Wilk et al. fabricated flexible PSCs with a 1 cm^2^ active area using a green solvent mixture (γ‐Butyrolactone (GBL), 2‐Methylpyrazine (2MP), and DMSO), achieving a PCE of 11.4% [19]. Although GBL is less toxic than DMF and N‐methyl‐2‐pyrrolidone (NMP), it is classified as a psychoactive substance and faces legal restrictions in several countries [20, 21]. Alternatives such as GVL, a biomass‐derived, eco‐friendly solvent, has been explored by Miao et al., demonstrating a PCE of 25.09% in spin‐coated FAPbI_3_ [22]. Kim et al. reached 20.6% in spin‐coated FAPbI_3_ by incorporating methylammonium chloride (MACl) to improve GVL solubility [23], while Chalkias et al. achieved 13.07% in inkjet‐printed MAPbI_3_, using GVL [18]. But most of these studies focus on narrow‐bandgap (NBG) perovskites < 1.6 eV. To enable TSC architectures, which require wide‐bandgap (WBG) perovskites, further research is needed.

When a scalable fabrication technique like inkjet printing is combined with green solvents to fabricate WBG PSCs, three key challenges, such as solubility, printability, and wettability, must be addressed. Achieving high‐quality perovskite thin films by inkjet printing requires precise control over the solvent composition. This involves dissolving all precursor materials in a specially formulated ink, which can then be printable and form stable droplets [24, 25, 26]. Specifically, WBG compositions contain significant amounts of bromide (Br^−^) and cesium (Cs^+^) ions, which pose solubility challenges [27, 28]. Also, interactions between the substrate surface and the ink should be optimized either through surface treatments or by adjusting the layer stacks to ensure good wettability [29, 30]. Additionally, for high‐performance PSCs, precise control over nucleation and crystallization of the deposited perovskite wet films is crucial. Typically, spin‐coated perovskite thin films are post‐treated with anti‐solvent quenching to regulate crystallization. However, most of the anti‐solvents are toxic, and this technique is not suitable for large‐area perovskite deposition. Vacuum quenching, gas quenching, air‐knife blade drying, and laser annealing emerge as feasible alternatives that support sustainable perovskite fabrication [31, 32]. Overcoming these issues is essential for developing efficient, sustainable inkjet‐printed PSCs. To date, the problem of eliminating the toxic solvents in the fabrication of inkjet‐printed WBG PSCs has not been completely solved.

In response to these challenges, this work establishes a comprehensive framework for sustainable, scalable inkjet printing of WBG perovskite photovoltaics. We introduce a triple‐cation, mixed‐halide WBG perovskite ink formulated in the green solvent γ‐valerolactone (GVL). Importantly, it is not self‐evident that GVL, although previously reported, can be successfully adapted as a solvent system for inkjet‐printed perovskite absorbers. Inkjet printing imposes markedly different constraints, including viscosity, surface tension, volatility, and jetting stability, which necessitate a dedicated formulation strategy. We demonstrate that the optimized GVL:DMSO ink exhibits favorable rheological properties for stable droplet ejection and clogging‐free inkjet printing. Interfacial wettability and drying dynamics are systematically controlled through surface modification with functionalized silica nanoparticles, suppressing drying patterns and ensuring uniform film formation. Crystallization quality and surface morphology are further enhanced by incorporating MACl, while bilayer surface passivation reduces non‐radiative recombination and boosts device performance. Building on these advances, we integrate the inkjet‐printed wide‐bandgap perovskite absorber into perovskite/silicon tandem architectures, achieving efficiencies exceeding 28%. Together, these results demonstrate a viable pathway for high‐efficiency tandem photovoltaics manufactured via green solvent, one‐step inkjet printing, establishing a compelling platform for sustainable large‐area PSC production.

## 2 Engineering Inkjet‐Printable Non‐Toxic Solvent System for Wide‐Bandgap Perovskite

To demonstrate the potential of inkjet‐printed green solvent‐based perovskite, it is essential to develop a printable precursor system that meets both sustainability and printing requirements. In this section, we detail the engineering of non‐toxic WBG perovskite inks tailored for inkjet printing. This development is presented in two key steps. The evaluation of solvent toxicity and perovskite ink printability, together with stable droplet ejection, is addressed.

### 2.1 Toxicity Evaluation

We begin by assessing the toxicity of solvents used in the processing of perovskite ink. The choice of solvents plays a critical role in PSC processing as it influences the ink formulation, film morphology, device performance, and environmental sustainability [33]. The definition and classification of green solvents remain a subject of debate, as they may depend on various factors and their usage. To categorize solvents, particularly in the context of perovskite film fabrication, the environmental and human health impacts of these solvents are evaluated. This assessment is based on previous studies [32], General Hazard Classifications (GHS), and data from Material Safety Data Sheets (MSDS). In this study, the greenness of a solvent is studied based on four key factors: Health Hazard Index, Environmental Hazard Index, Toxicity Index, and Safety Index. The Environmental Hazard Index measures the solvent's biodegradability and its toxicity to aquatic life. The Health Hazard Index assesses the general risks posed to users, considering whether the solvent is acutely toxic (e.g., skin irritation or eye irritation) or is classified as a CMR (carcinogenic, mutagenic, or reprotoxic) material. The Toxicity Index evaluates exposure limits based on lethal dosage (LD50) values, while the Safety Index considers physical properties such as flammability, reactivity, and stability of the material. Frequently used polar aprotic solvents in perovskite research are selected, and detailed information on these indices for each solvent is compiled in Tables S1, S2, S3, and S4. Based on the evaluation of these four indices, the solvents are categorized by impact level (low, medium, or high) and listed in Figure S1. Traditionally, high‐polarity amide‐based solvents such as DMF, NMP, and dimethylacetamide (DMAc) are widely used due to their excellent solubility for lead halide precursors. However, DMF, DMAc, NMP, and 1,3‐Dimethyl‐2‐imidazolidinone (DMI) are classified as category 1B reproductive toxic solvents, and DMF is also associated with hepatotoxicity [34, 35, 36, 37]. Additionally, they can cause potential carcinogenic effects upon prolonged exposure. Some of the solvents mentioned also exhibit high volatility and dermal absorption rates, posing occupational health risks during device fabrication [38, 39]. From the data collected, it is evident that the Toxicity Index and Health Hazard Index for solvents such as DMF, DMAc, and NMP are among the highest, necessitating the use of controlled handling and stringent safety measures. Acetonitrile and tetrahydrofuran (THF) are not high health hazards, but they have high vapor pressures that can accelerate solvent evaporation [40]. Acetonitrile can also lead to cyanide formation when used for prolonged periods at higher temperatures [38]. This leads to potential inhalation risks when processed in ambient air. DMI and THF exhibit poor biodegradability, resulting in long‐term environmental accumulation. Compared to the previously mentioned solvents, GBL is greener. However, it has strict legal restrictions in some countries as it is classified as a psychoactive material [21, 41]. DMSO, GVL, Ethanol, and Isopropanol (IPA) emerge as some of the green alternatives to fabricate environmentally sustainable PSCs [42, 43, 44] Compared to other solvents, GVL exhibits superior environmental credentials due to its sustainable production pathway. Derived from lignocellulosic biomass, GVL is synthesized through a fully renewable process, enhancing its appeal as a green solvent [45]. This makes GVL a promising candidate for environmentally friendly perovskite fabrication, aligning with the principles of green chemistry and sustainable processing of PSCs.

### 2.2 Ink Formulation

Second, we examine the ink formulation to achieve a stable, printable ink that dissolves the precursor materials required for the perovskite formulation. GVL, a bio‐derived solvent, is used to dissolve all the precursor salts (cesium iodide (CsI), formamidinium iodide (FAI), methylammonium bromide (MABr), lead bromide (PbBr_2_), and lead iodide (PbI_2_)) to fabricate a triple cation mixed halide perovskite. Dissolving lead halide precursors requires solvents with high donor numbers, which reflect their Lewis basicity and ability to coordinate with Pb^2^^+^ ions [46]. DMF, commonly used to dissolve perovskite precursors, has a donor number of 27 kcal mol^−1^, enabling strong Pb^2+^ interactions. In contrast, GVL's lower donor number (18.1 kcal mol^−1^) limits its solvation capability [23]. In our experiments, using pure GVL resulted in early crystallization, as evidenced by the formation of a black phase in the vial (Figure 1A).

**FIGURE 1:**
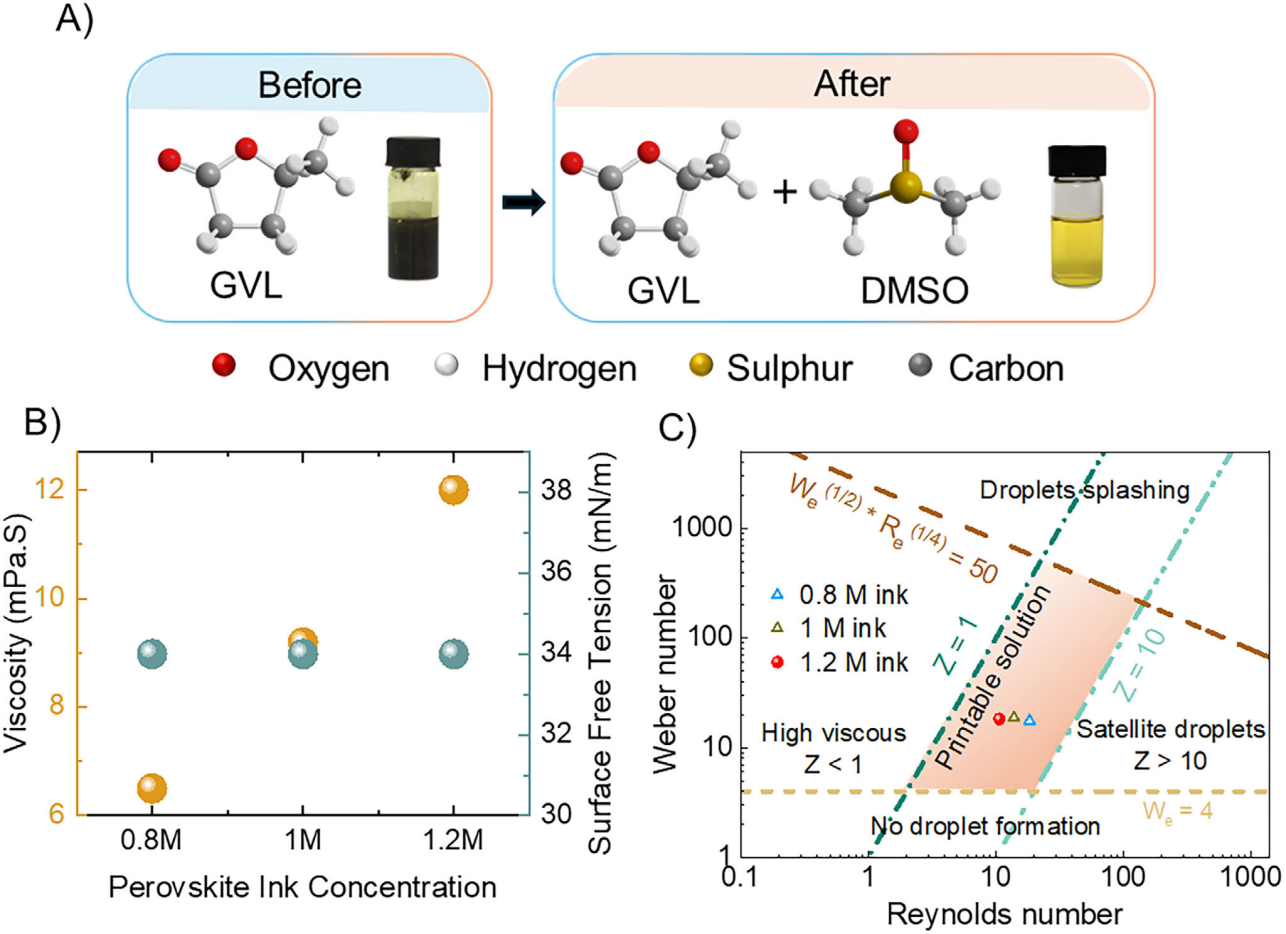
(A) Solubility of the triple cation double halide perovskite with GVL and mixture of GVL: DMSO, (B) Concentration‐dependent rheological properties of the perovskite ink, (C) Printability graph: Weber number plotted against the Reynolds number, with the region where the inverse Ohnesorge number falls between 1 and 10 (1 < Z < 10) highlighted in orange. The developed triple cation double halide perovskite ink (1.2 m), represented by a red dot, is within the printability zone.

Solvents with low donor numbers tend to promote iodoplumbate formation, increasing the risk of premature crystallization in perovskite inks [23]. To prevent this, a strong coordinating co‐solvent is needed. DMSO, with a high donor number (∼29.8 kcal mol^−1^) and green solvent status, forms stable PbI_2_‐DMSO complexes that suppress early crystallization by reducing free Pb^2+^ [47] resulting in a stable ink (Figure 1A). In this study, we address GVL's limited solubility by introducing DMSO as a co‐solvent. Solvent ratios and ink concentration are also optimized to control surface tension and viscosity, which are key parameters for stable inkjet droplet formation [48].

Concentrations beyond 1.2 м are not tested, since the solubility of the ink decreased with increased concentration (Figure S2). Figure 1B illustrates the dependence of ink rheology and surface properties on perovskite concentration (0.8 m, 1 m, 1.2 m). The viscosity rises from 6.5 mPa·s at 0.8 m to 12 mPa·s at 1.2 m. By contrast, the surface free tension remains nearly constant (34 mN m^−1^) across the concentration range, suggesting that interfacial properties changed primarily by the solvent system rather than the perovskite precursors. This decoupling of bulk viscosity and surface tension indicates that concentration primarily modulates flow and mass transport during film deposition while preserving wetting behavior.

### 2.3 Printability

An increase in concentration of the solution is found to correlate with higher ink density and viscosity, as shown in Figure 1B. Specifically, increasing the concentration from 0.8 to 1.2 m results in an absolute increase in the viscosity of 5.5 mPa·s. To ensure the developed GVL‐based perovskite ink is suitable for inkjet printing, its printability is evaluated using the Reynolds number (Re) and the Weber number (W_e_). These dimensionless numbers are critical in determining the ink's properties during the printing process. The Reynolds number (R_e_) is defined as the ratio of inertial forces to viscous forces within the fluid [R_e_ = νρa/η]. The Weber number (W_e_) represents the ratio of inertial forces to surface tension forces [W_e_ = ν^2^ρa/γ], where, ν: velocity of droplets, η: viscosity of the perovskite ink, ρ: density of perovskite ink, γ: surface tension of the ink, a: diameter of the printhead cartridge nozzle. A high R_e_ indicates that inertial forces dominate over viscous forces, which is essential for stable droplet formation in inkjet printing. A suitable W_e_ in the range of 2 < W_e _< 25 ensures that the ink can form droplets without breaking up prematurely or forming satellite droplets. By plotting the R_e_ and W_e_ values, the printability region of our ink is identified. For stable and precise inkjet printing, the ink must fall within the optimal printability window, often defined by the parameter Z (Ohnesorge inverse). Z is a dimensionless number, defined as the ratio of the R_e_ to the square root of the W_e_ [Z  =  1/O_h_  =  *R_e_*/$\sqrt{W_{e}}$ = $\sqrt{\gamma \rho a}\;$/η]. It is independent of the ink velocity [49]. To obtain stable droplets, the Z value should lie between 1 and 10 [33, 49]. The calculated Z value for 0.8, 1, and 1.2 m GVL‐based inks is within the printable region, showing Z  =  4.4, 3.2, and 2.5, respectively. This indicates that the developed ink possesses the necessary fluid properties for stable and precise inkjet printing (Figure 1C). To achieve thicker perovskite films without increasing the solvent proportion, a concentration of 1.2 m is selected as the optimal ink formulation for this study.

## 3 Controlling Wetting and Drying Artifacts in Inkjet‐Printed Perovskite Films

Upon developing a printable green perovskite ink, its wettability on the hole transport layer (HTL) is evaluated to ensure uniform thin‐film formation during inkjet printing. Figure 2A,B illustrate the complete PSC device architecture and the corresponding inkjet‐printing process used in this work. The stack consists of Glass/ITO/2PACz/functionalized silica nanoparticles (SiO_2_‐NP)/WBG inkjet‐printed perovskite/passivation layer/C_60_/Bathocuproine (BCP)/silver. Beyond printability, the wettability of the ink on the layer beneath remains a critical factor influencing thin film uniformity and device performance [50].

**FIGURE 2:**
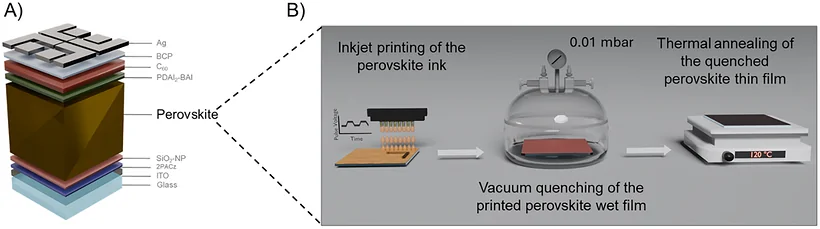
(A) Schematic illustration of the developed perovskite solar cell with p‐i‐n stack, (B) Fabrication of inkjet‐printed perovskite thin film, (i) Deposition of perovskite ink using drop‐on‐demand inkjet printing, (ii) As‐deposited perovskite wet film, (iii) Post‐treatment of perovskite wet film using vacuum quenching followed by thermal annealing.

### 3.1 Wettability

The effect of SiO_2_‐NP incorporation on the interfacial properties and film formation behavior of Glass/ITO/2PACz substrates was examined by correlating morphological evolution, wetting characteristics, and surface free energy (SFE). As shown in Figure 3A,B, the pristine 2PACz film (0 wt.% SiO_2_‐NP) exhibits pronounced macroscopic pinholes. Microscale inspection reveals complex wrinkling in the perovskite thin film, indicating that the 2PACz surface lacks the rheological properties required for good wetting during the inkjet printing process. Correspondingly, the measured contact angle is relatively high (∼34°), confirming the hydrophobic nature of the unmodified surface (Figure 3C).

**FIGURE 3:**
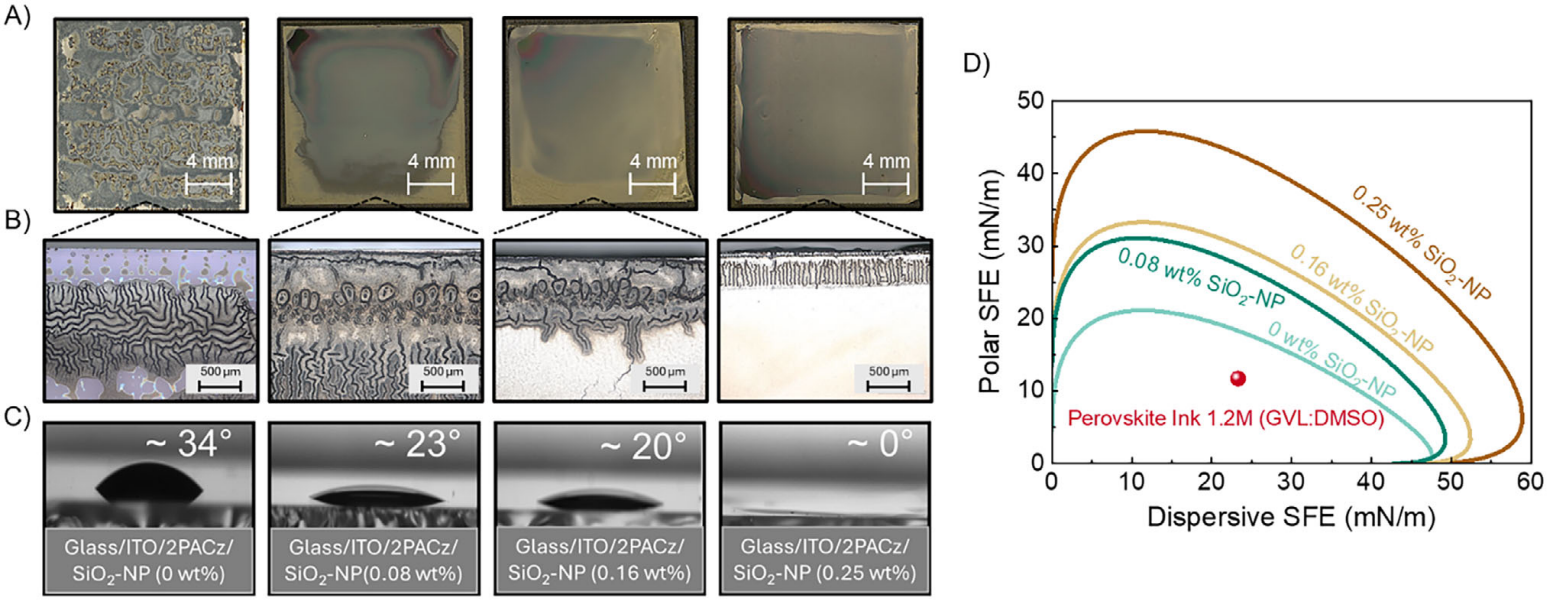
(A) Drying patterns (B) Microscopic images close to the edges, and (C) Contact angle of the 1.2 м perovskite ink on the perovskite thin film deposited on the hole transport layer with different silica nanoparticle concentrations and their corresponding contact angles, (D) Wetting envelope (at 0° contact angle plotted by considering polar and dispersive parts of surface free energy) of thin films with various concentrations of SiO_2_‐NP.

With the addition of SiO_2_‐NP (0.08‐0.16 wt.%), the severity of these drying patterns is significantly reduced. Although residual wrinkling persists, the amplitude, spacing, and disorder of the patterns decrease, suggesting that the nanoparticles help dampen the wetting issue by promoting more uniform drying. The gradual reduction in contact angle (from ∼23° at 0.08 wt.% to ∼20° at 0.16 wt.%) confirms that progressive SiO_2_‐NP incorporation enhances surface hydrophilicity, improving wetting behavior and assisting in the formation of smoother thin films. At a 0.25 wt.% concentration of SiO_2_‐NP, the thin films become remarkably uniform, with negligible visible macroscopic or microscopic patterning. The contact angle approaches 0°, indicative of complete wetting. We assume that functionalizing SiO_2_‐NP with amine groups increases their surface polarity. The amine group can enhance interactions between the surface and other materials, thereby improving wetting and adhesion [51]. Torkhani et al. investigated the effects of amino‐treated SAMs and found that the amino group improved the compatibility of the perovskite layer, leading to better morphology [52].

This qualitative trend is quantitatively confirmed by the surface free energy analysis (Figure 3D). The SFE plots show that increasing the SiO_2_‐NP concentration leads to a substantial increase in the polar component of the SFE, while the dispersive component changes more modestly. The SFE envelope expands significantly at 0.25 wt.%, demonstrating the formation of a highly polar, energetically active surface. This enhanced energetic compatibility facilitates improved wetting and uniform spreading during perovskite deposition. The observed suppression of drying instabilities, the establishment of a fully wetting interface, and the increased polar SFE component collectively indicate that nanoparticle incorporation transforms the 2PACz surface into a more favorable scaffold for solution‐processed perovskite thin films.

We now evaluate the impact of wettability control on device performance. Devices printed with 0.08 wt.% SiO_2_‐NP show significant variation in *J*_SC_ (Figure S3), attributed to inhomogeneous drying and coffee‐ring effects during vacuum quenching (Figure 3A,B). These effects arise from capillary flows that transport solutes to the droplet edges due to uneven evaporation. Suppressing this requires inducing Marangoni flow, which can be achieved by slowing down evaporation [53]. Increasing the SiO_2_‐NP concentration to 0.25 wt.% enhances Marangoni flow by creating surface‐tension gradients, thereby reducing the coffee‐ring effect and improving thin film uniformity. This concentration also maintains PSC performance and reproducibility, while higher concentrations are avoided due to reported PCE losses associated with the insulating nature of SiO_2_‐NP [54]. At 0.25 wt.%, coffee‐ring effects are minimal, and short‐circuit current (*J*_SC_) variation is significantly reduced (Figure 3A,B and Figure S3).

While substrate pre‐treatment influences drying patterns, post‐treatment is equally critical for achieving uniform, well‐crystallized perovskite thin films. Solvents with low vapor pressure and high boiling points prolong evaporation, allowing solutes to migrate to the thin film edges and form coffee‐ring patterns, which degrade thin film quality and PSC performance [53]. In the conventional spin coating process, anti‐solvent quenching followed by annealing rapidly removes solvents and suppresses such effects. To avoid toxic anti‐solvents and enable scalability, we employ vacuum quenching. By lowering the solvent's boiling point, vacuum conditions accelerate evaporation and reduce solute redistribution [55]. In our process, printed wet films are quenched at ∼1 × 10^−2^ mbar. However, quenching time must be optimized: prolonged exposure increases surface roughness (Figure S4A), while overly rapid quenching can cause macro‐cracks (Figure S4B). A 60 s quenching time yields uniform δ‐phase films (Figure S4C), which convert to the photoactive black phase upon annealing. At 150°C, residual PbI_2_ increases, indicating degradation, whereas at 120°C, it is minimized and thin film quality is preserved (Figure S5). Thus, 120°C is identified as the optimal annealing temperature for producing highly crystalline, uniform films essential for high‐performance devices.

## 4 Additive and Interface Engineering for Wide‐Bandgap Perovskite Solar Cells

Following the deposition of a homogeneous perovskite thin film, the focus shifts to additive engineering and surface passivation strategies to improve PSC performance. MACl is incorporated into the perovskite solution to enhance the crystallinity and morphology of the inkjet‐printed perovskite thin films. Using x‐ray Diffraction (XRD), we identify an increased crystallinity upon MACl introduction. XRD patterns reveal that the perovskite thin films without MACl exhibit two similarly strong intensity peaks at 12° and 14° diffraction angles, corresponding to the (001) reflection of PbI_2_ and (100) reflection of the perovskite (Figure 4A). In contrast, perovskite thin films with MACl show an enhanced intensity of the (100) reflection peak. This suggests higher crystallinity and enhanced phase purity, along with a reduction in the remaining unconverted PbI_2_ [56]. The unreacted PbI_2_ crystals can decompose into lead and iodide within the perovskite thin films, hastening the degradation under illumination [57]. The addition of MACl therefore facilitates perovskite conversion, yielding a higher‐quality absorber layer.

**FIGURE 4:**
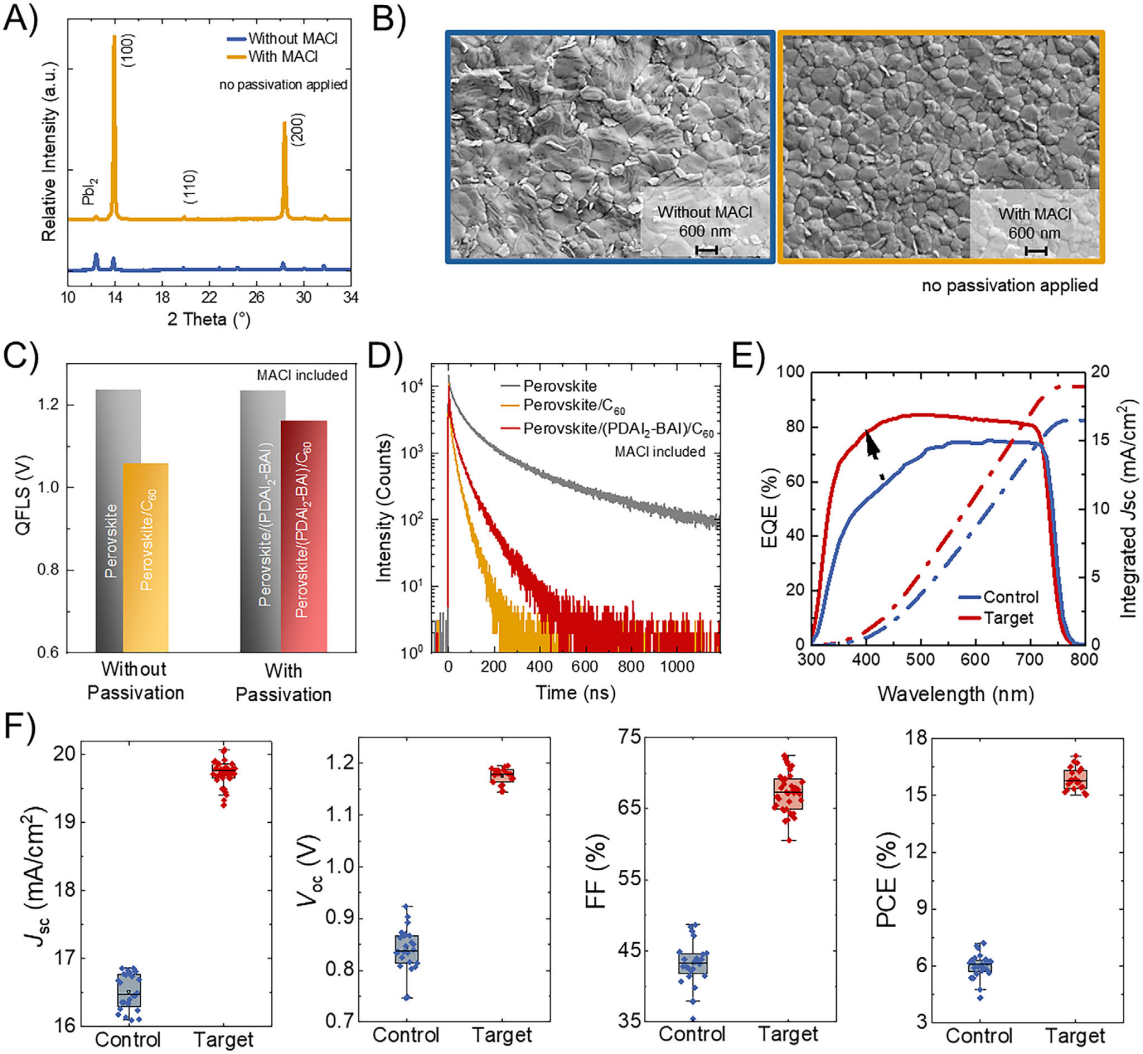
(A) X‐ray Diffraction pattern of the perovskite thin film without and with MACl, (B) Top‐view SEM of perovskite thin films without (blue) and with MACl (yellow), (C) Calculated QFLS values from absolute photoluminescence measurements for passivated and non‐passivated perovskite thin films, (D) Time‐resolved spectroscopy measurements for perovskite thin film with and without passivation, (E) External Quantum Efficiency and calculated integrated current density (*J*_SC_) of the control and target (MACl and (PDAI_2_‐BAI) passivation) perovskite solar cells, (F) Photovoltaic parameters of control and target (MACl and (PDAI_2_‐BAI) passivation) perovskite solar cells.

Scanning electron microscopy (SEM) images reveal morphological changes in perovskite thin films upon treatment with MACl. Perovskite thin films without MACl exhibited a rough surface with numerous micrometer‐sized wrinkles (Figure S6). However, when MACl is added to the perovskite precursor, the wrinkles disappear, resulting in a smooth perovskite thin film. Previous studies suggest that the surface morphology of perovskite thin films is influenced by evolving stress during post‐treatment of the thin film [58]. The initial transition from the δ‐phase to the α‐phase can introduce defects due to the rapid structural change from the intermediate phase to the perovskite phase, which involves ionic movement between adjacent [PbI_6_]^4−^ octahedra [59]. This transition can potentially lead to the formation of wrinkles [58]. Adding MACl reportedly slows the crystallization of the perovskite. This allows stress to be relieved during grain growth, thereby preventing wrinkle formation [58, 60]. Furthermore, the addition of MACl results in grains being more systematically arranged, creating a homogeneous structure (Figure 4B). In contrast, perovskite thin films without MACl appear not to be fully crystallized, with numerous micrometer‐sized agglomerations distributed on the thin film's surface (Figure 4B). This can explain the increase in the XRD peak at 14° diffraction angle for the films with MACl, suggesting improved perovskite crystal quality. Enhanced crystallinity and enlarged grain size mitigate these defects within the bulk and at grain boundaries, as supported by XRD and SEM analyses. The suppression of trap states correlates with the increase in *J*_SC_, as fewer recombination centers allow for more efficient charge extraction [61].

Building on this optimized perovskite bulk structure, the effect of the surface passivation, in controlling interfacial carrier dynamics, is obtained through steady‐state photoluminescence (PL) and time‐resolved photoluminescence (TRPL) measurements. In this case, perovskite thin films incorporated with MACl are used as a baseline measurement and compared with two configurations: with and without (PDAI_2_‐BAI) bilayer passivation. The combined quasi‐Fermi level splitting (QFLS) and TRPL measurements reveal profound differences in interfacial recombination dynamics between the perovskite thin films with and without passivation (Figure 4C,D). For the bare perovskite thin film, an amplitude‐averaged lifetime of ∼60 ns is obtained (Figure S7), reflecting the intrinsic recombination behavior in the absence of interfacial quenching. Upon deposition of C_60_ directly onto the perovskite thin film, the QFLS is significantly reduced compared to the bare perovskite, indicating that the electron‐extracting interface acts as a significant non‐radiative recombination sink (Figure S8). These losses are typically associated with energetic disorder and interfacial defect states, which act as recombination centers for charge carriers [62]. Consistent with this interpretation, TRPL measurements show that the non‐passivated sample exhibits a rapid decay within the first 100–200 ns, with the lifetime significantly reduced to ∼13 ns, indicating fast carrier quenching driven by interfacial trap states. In striking contrast, the sample with passivation, employing the (PDAI_2_‐BAI) interlayer before C_60_ deposition, preserves a substantially higher QFLS, approaching the intrinsic radiative limit of the perovskite. This enhancement reflects the suppression of interfacial non‐radiative pathways that are typically activated during C_60_ deposition. The TRPL data correlates to this observation: the passivated sample exhibits a partially recovered lifetime of ∼17 ns, alongside a noticeably slower decay profile. The decay kinetics shift from a strongly trap‐dominated regime toward a more radiative recombination process, indicating that the interlayer effectively passivates defect sites while maintaining efficient charge‐carrier extraction. Together, these results demonstrate that the (PDAI_2_‐BAI) surface modification fundamentally alters the interfacial physics at the electron‐selective contact. By reducing the interfacial trap density and promoting selective carrier extraction without sacrificing radiative quality, the target stack achieves a substantially higher QFLS and prolonged carrier lifetime, two key indicators of minimized non‐radiative loss [61, 63, 64, 65]. Such improvements directly translate into an interface approaching the Shockley‐Queisser radiative limit, thereby enabling higher *V*_OC_ and improved photovoltaic performance.

The synergistic effects of bulk additive engineering and interface passivation are ultimately reflected in the photovoltaic device performance. Incorporating MACl in the perovskite bulk, together with a (PDAI_2_‐BAI) bilayer passivation on top of the perovskite, leads to a substantial increase in PCE from 8% in control PSCs to 17.5% in target PSCs. The external quantum efficiency (EQE) measurements reveal that the target PSCs with MACl and (PDAI_2_‐BAI) exhibit a higher integrated *J*_SC_ across a broad spectral range than the control devices without MACl and (PDAI_2_‐BAI) (Figure 4E). The EQE of the champion device demonstrates efficient charge carrier extraction. The integrated *J*_SC_ derived from the EQEs is 19 mA cm^−2^ for the target PSC and 16.51 mA cm^−2^ for the control PSC. This overall performance improvement arises from enhancements in *J*_SC_ (17 to 20 mA cm^−2^), open‐circuit voltage (*V*_OC_) (0.95 to 1.2 V), and fill factor (FF) (48% to 72%) as supported by the statistical analysis (Figure 4F, and Figure S9, and Table S5). This combined strategy enables efficient charge generation and extraction, ultimately leading to high‐performance perovskite solar cells.

## 5 Inkjet‐Printed Perovskite in Perovskite/Silicon Tandem Solar Cell

To assess the potential of the developed WBG PSCs in tandem architectures, two‐terminal TSCs are fabricated. These TSCs incorporate the optimized green solvent based inkjet‐printed perovskite layer in PSC as the top sub‐cell, paired with a compatible silicon bottom cell to extend spectral utilization and enhance overall energy conversion, resulting in a tandem architecture: silicon bottom cell (Qcells industrial cell technology)/ITO/Ph4PACz/SiO_2_‐NP/inkjet‐printed perovskite absorber/(PDAI_2_‐BAI)/C_60_/SnO_2_/IZO/Ag/MgF_2_, (Figure 5A) with an active area of 1.0 cm^2^. The SEM images of the inkjet‐printed perovskite thin film deposited onto the silicon bottom cell are shown in Figure 5A. In the cross‐sectional view, the inkjet‐printed perovskite thin film shows columnar grains and minimal visible defects. The top‐view image shows grain sizes ranging from 800 nm to 1 µm. Such large grains contribute to a reduced grain boundary surface area, which is beneficial for minimizing defect densities in the perovskite thin films (Figure 5A). The *J*–*V* measurement of the champion TSC demonstrates a PCE of 28.2%, with a *V*_OC_ of 1.94 V, and a *J*_SC_ of 19.25 mA cm^−2^, indicating effective photogeneration and voltage addition from the two sub‐cells (Figure 5B). The stability test is performed on unencapsulated perovskite/silicon TSCs. The TSC retains 80% of its PCE after 190 h of continuous illumination (Figure 5C). EQE measurements confirm that the integrated *J*_SC_ aligns with the value obtained from *J–V* characterization. Both sub‐cells show a current mismatch of 1.5 mA cm^−2^, with perovskite yielding 19.2 mA cm^−2,^ and silicon sub‐cell 20.7 mA cm^−2^. The overall current is limited by the top cell (Figure S10). Nevertheless, the TSCs exhibit an FF of only 75.05%, which is the primary performance limitation. Despite these limitations, the results serve as proof of concept, demonstrating the feasibility of integrating the developed WBG PSCs into a tandem configuration. These findings underscore the need for further optimization in interfacial engineering and current matching strategies. However, using these techniques, a tandem configuration with PCE exceeding 28% has been achieved, representing the first demonstration of a fully printed perovskite in tandem architecture processed entirely using green solvents (Figure 5D and Table S6).

**FIGURE 5:**
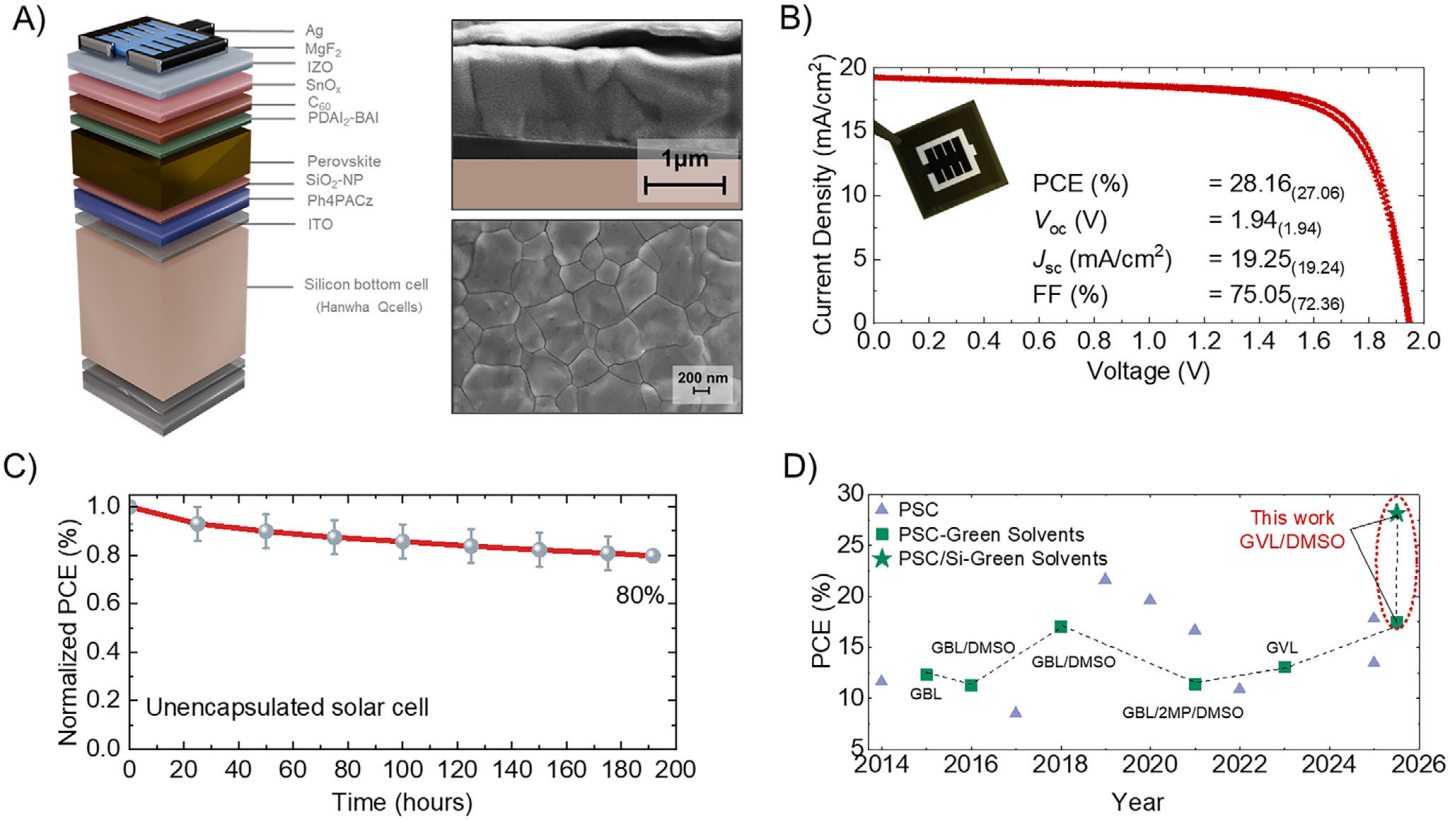
(A) Sketch of the tandem solar cell architecture, cross‐sectional and top‐view SEM images of the inkjet‐printed perovskite thin film in tandem architecture, (B) Current density‐voltage (*J–V*) characteristics of the champion monolithic perovskite/silicon tandem solar cell, where the perovskite top cell is fabricated using green solvents and inkjet printing, (C) Maximum power point tracking of the tandem solar cell over 190 h under continuous illumination without encapsulation, (D) Comparison of the power conversion efficiencies of perovskite solar cells fabricated with inkjet printing. Efficiencies from this work are highlighted under red circle. The comparison does not consider solar cell area, architecture, or bandgap.

## 6 Conclusion

To advance the commercialization of solution‐processed PSCs, it is crucial to address hazardous solvents, particularly in scalable fabrication processes. This study demonstrates the successful development of efficient inkjet‐printed PSCs using environmentally friendly solvents across all layers. By studying and categorizing the environment, health, safety, and toxicity indices of various polar aprotic solvents, the risks associated with perovskite fabrication are assessed. The limited solubility of the WBG perovskite precursors with lead halides and cesium in GVL poses a challenge in achieving a stable perovskite ink. However, introducing a co‐solvent, DMSO, with a high donor number, enables the formation of PbI_2_.complexes, thereby delaying perovskite crystallization and producing a stable ink. The ink concentration is further optimized to ensure solubility and printability. Finally, a 1.2 м solution with a mixture of green solvents, GVL, and DMSO is characterized for printability, with the Z value within the printable range. Wetting and drying on the substrate are optimized by tuning the interaction between the surface and the ink. An amino‐functionalized SiO_2_‐NP layer is introduced to enhance the substrate's surface free energy, thereby improving its wettability. Incorporating MACl into the bulk of the perovskite effectively improves its crystallinity and morphology, thereby increasing *J*_SC_. Lastly, by introducing PDAI_2_‐BAI bimolecular passivation, the surface recombination at the perovskite‐C_60_ interface is reduced. With both strategies, the green solvent‐based inkjet‐printed WBG single‐junction PSCs and perovskite/silicon TSCs achieved PCEs above 17% and 28%, respectively. This strategy offers a sustainable pathway to the commercialization of efficient, scalable photovoltaics.

## 7 Experimental Methods

### 7.1 Materials

All materials are used as received without any further modifications. MABr (99.99% – GreatCell Solar), FAI (99.99% – GreatCellSolar), CsI (99.99% – TCI chemicals), PbBr (99.99% – TCI chemicals), PbI_2_ (99.99% – TCI chemicals), 2PACz (Luminescence Technology Corp.), Silica nanoparticles (nanoComposix – 20 nm Aminated), N, N‐dimethylsulfoxide (99% – Sigma–Aldrich), γ‐Valerolactone (Sigma–Aldrich), MACl (99.5% – Luminescence Technology Corp.), PDAI_2_ and BAI (GreatCell Solar), Ethanol (>99.8% – VWR Chemical), Isopropanol (>99.8% – VWR Chemical), C_60_ (Sigma–Aldrich), BCP (Lumtec), Methanol (>99.9% – VWR Chemical), (4‐(3,6‐diphenyl 9H‐carbazol‐9‐yl)butyl)phosphonic acid (Suzhou LiWei Tech Co., Ltd).

### 7.2 Perovskite Solar Cell Fabrication

#### 7.2.1 Single‐Junction PSC

To fabricate the single‐junction PSCs, glasses sputtered with patterned indium tin oxide (ITO) (120 nm, 15 Ω) from Luminescence Technology Corp (Lumtec) were used. In the next step, these substrates were cleaned in a deionized water ultrasonic bath for 15 min, followed by an acetone ultrasonic bath for 15 min, and then in an isopropanol ultrasonic bath for 15 min. The substrates were dried using a nitrogen gun and then treated with 100% oxygen plasma for 15 min. Then, the HTL, 2PACz was spin‐coated at 3000 rpm (1000 rpm s^−1^, 30 s). 0.5 mg mL^−1^ of 2PACz was dissolved in ethanol, and the solution was left for 60 min under ultrasonication before deposition. After deposition, the substrates were annealed on a hot plate at 100°C for 10 min. Afterward, ethanol‐based SiO_2_‐NP were diluted according to the concentrations used in this study. SiO_2_‐NP were deposited using spin coating (2000 rpm, 1000 rpm s^−1^, 20 s) and then annealed at 100°C for 10 min after deposition. Next, the 1.2 m perovskite ink for inkjet printing is prepared by dissolving FAI: 151 mg mL^−1^, MABr: 31 mg mL^−1^, PbBr: 109 mg mL^−1^, MACl: 32 mg mL^−1^, PbI_2_: 470 mg mL^−1^ in a 4:1 ratio of GVL and DMSO. The solution was prepared one day before printing and allowed to stir at 60°C, 600 rpm. Additionally, 1.2 m (CsI: 312 mg mL^−1^) was dissolved in DMSO, and 55 µL of CsI/DMSO solution was mixed separately into the above‐dissolved perovskite solution on the day of printing to achieve a 1.68 eV bandgap with a composition of FA_0.73_MA_0_._22_Cs_0.05_Pb(I_0.77_Br_0_._23_)_3_. The perovskite solution was then filtered using a 0.2 µm PTFE filter to remove any undissolved particles or impurities before printing. For inkjet printing of the perovskite thin film, a Meyer Burger (now SÜSS MicroTec) PiXDRO LP50 inkjet printer with a 2.4 pl DMC Samba Fujifilm printhead is used. The perovskite ink is inkjet‐printed at an optimized resolution of 1600dpi at a print speed of 150 mm s^−1^. The volume of the droplet is optimized to ∼ 2.5 pL with a double‐pulse waveform (40 V voltage – 2.5 µs width – 4 µs space: 40 V voltage – 20 µs width – 5 µs space). The head temperature during the printing is set to 35°C, and the ink pressure was set to −30 mbar. The total area of the inkjet‐printed perovskite thin film is 15 × 15 mm^2^ per substrate. Afterward, the inkjet‐printed thin films were manually transferred to a vacuum chamber (Vacuumtechnik Vertriebs GmbH), where the layers were quenched by evacuating the chamber to 2 ^*^ 10^−2^ mbar pressure for 1 min, followed by thermal annealing at 120°C for 15 min. The overall printing was done in ambient conditions (∼23°C, 45% relative humidity). After the deposition of the perovskite thin film, a surface passivation layer was spin‐coated inside a nitrogen glove box. PDAI_2_ (1.25 mg mL^−1^) was dissolved in isopropanol and ultrasonicated for about 24 h before deposition. BAI (1.25 mg mL^−1^) was dissolved in isopropanol alcohol separately and ultrasonicated for 10 min. Before depositing, in a ratio of 1:1, both PDAI_2_ and BAI solutions were mixed and spin‐coated (4000 rpm, 1000 rpm s^−1^, 30s), followed by annealing at 100°C for 10 min. The electron transport layer (C_60_, 20 nm, 0.2 Å/s) and an interfacial buffer layer (BCP, 5 nm, 0.2 Å s^−1^) were vapor‐deposited using a thermal evaporator from Kurt J. Lesker. After evaporation, the ITO contacts were re‐exposed by scratching the substrate edges with a scalpel. Subsequently, silver (100 nm, 0.5 Å s^−1^) was deposited through a mask via thermal evaporation using a VacTec COAT340 evaporator. This process resulted in the creation of four PSCs on each substrate (each measuring 10.5 mm^2^).

#### 7.2.2 Perovskite/Silicon Tandem Solar Cell

The silicon bottom cells were cleaned with acetone and IPA via spin‐coating. For the fabrication of TSCs, the processing steps up to the deposition of the C_60_ layer were similar to those employed for the single‐junction PSCs, except for the HTL and passivation. For the HTL, a SAM molecule, (4‐(3,6‐diphenyl‐9H‐carbazol‐9‐yl)butyl)phosphonic acid (0.5 mg mL^−1^) dissolved in methanol was deposited by spin‐coating with the same recipe of 2PACz. For the passivation, PDAI_2_ (0.3 mg mL^−1^) was dissolved in isopropanol and ultrasonicated for about 24 h before deposition. BAI (0.3 mg mL^−1^) is dissolved in isopropanol alcohol separately and ultrasonicated for 10 min. Before depositing, in a ratio of 1:1, both PDAI_2_ and BAI solutions were mixed and spin‐coated (4000 rpm, 1000 rpm s^−1^, 30s), followed by annealing at 100°C for 10 min. Later, following the C_60_ layer, a 20 nm SnO_2_ buffer layer was deposited by ALD. During ALD deposition, the substrate temperature was maintained at 90°C, with the Tetrakis(dimethylamino)tin(IV) (TDMASn) precursor held at 70°C and the H_2_O source at room temperature. The TDMASn pulse and purge times were 1 and 10 s, respectively, using 90 sccm N_2_, while for H_2_O, the pulse and purge times were 0.2 and 15 s with the same N_2_ flow rate. A total of 200 ALD cycles were performed. Subsequently, a 45 nm‐thick IZO transparent conductive electrode was sputtered using a shadow mask at 190 W with pure Ar and O_2_ (1 mTorr). The device's active area (1 cm^2^) was defined by a thermally evaporated 300 nm Ag electrode (C‐shape with a 100 µm grid). Finally, to minimize reflection losses, 110 nm of MgF_2_ was evaporated as an anti‐reflective layer on top of the Ag electrode.

### 7.3 Characterizations

#### 7.3.1 J–V Measurement

To assess the characteristics of the PSCs, a xenon‐lamp‐based solar simulator (Newport Oriel Sol3A) with an AM1.5 G spectrum (1000 W m^−2^) was calibrated using a certified silicon solar cell (Newport) equipped with a KG5 band‐pass filter. The PSCs were then measured in both backward and forward directions with a step increment of 10 mV. Measurements were taken from −0.2 to 1.2 V at a scanning rate of 0.6 V s^−1^ using a Keithley 2401 source measurement unit. The temperature of the PSCs was maintained at 25°C using a microcontroller‐adjusted Peltier element during the measurement process. The maximum power point (MPP) was tracked using a perturb‐and‐observe method. All measurements were conducted within a nitrogen glove box. The perovskite/silicon tandem solar cell was characterized with a class AAA LED‐based solar simulator from Wavelabs, LS‐2, with a scan rate of 0.6 V s^−1^ (Keithley 2400, AM 1.5G spectra, 100 mW cm^−2^). The measurements were conducted from −0.2 to 2 V, and they were characterized in ambient atmospheric conditions.

#### 7.3.2 External Quantum Efficiency Measurement

A PVE300 PV QE system (Bentham EQE system) was used to measure EQE. The calibration was performed with a silicon reference solar cell. Afterward, measurements were taken with a 0.74 mm^2^ illumination spot, a chopping frequency of 560–590 Hz, and an integration time of 500 ms. The step size for the measurements was 5 nm. All measurements were conducted within a nitrogen glove box. For perovskite/silicon TSCs characterization, measurements are taken at a step size of 10 nm. EQE was measured by “QE‐R Quantum Efficiency Measurement System from ENLITECH”. A wavelength range of 300–1200 nm was used. The system was calibrated by the certified silicon (Si) and germanium (Ge) reference cells for 300–1100 and 1000–1300 nm, respectively. The measurement was conducted in ambient atmospheric conditions.

#### 7.3.3 XRD Measurements

The crystal structure of the perovskite layers was analyzed using XRD (Bruker D2 Phaser system) with Cu‐Kα radiation (λ = 1.5405 Å) in Bragg–Brentano configuration, employing a LynxEye detector. The XRD measurement was performed on the perovskite thin film deposited onto the ITO/2PACz/SiO_2_‐NP substrate to replicate the same nucleation and crystallization conditions as those in the PSCs.

#### 7.3.4 SEM

SEM analysis was performed using a Zeiss LEO1530 SEM equipped with an in‐lens detector and an aperture size of 20–30 µm. The acceleration voltages applied for surface and cross‐sectional analysis varied between 3 and 5 kV.

#### 7.3.5 Viscosity, Contact Angle, SFE, and SFT

To calculate the wetting envelope and determine the polar and dispersive components of the substrate's SFE, the contact angle of perovskite ink on varying concentrations of Si‐NP was measured using the sessile drop method (Krüss DSA 100 drop shape analyzer system). Droplets (approximately 1–5 µL) were placed on the surface and measured after a brief settling period. The polar and dispersive SFE were computed using Owens‐Wendt‐Rabel‐Kaelble theory with a least absolute residual method. The dispersive and polar components of the surface free tension (SFT) of the inks were calculated from the total SFT, measured using the pendant drop method, and the contact angles measured on a PTFE substrate. Measurements were performed in ambient air conditions.

#### 7.3.6 Photoluminescence (PL)

Photoluminescence measurements were performed using a LuQY Pro system (Quantum Yield Berlin) equipped with an integrating sphere. A continuous‐wave laser (*λ* = 532 nm) was used for excitation. The system was spectrally calibrated for both excitation and emission, enabling detection of absolute photon flux. The sample absorbance at the excitation wavelength was accounted for within the analysis, and the quasi‐fermi level splitting (QFLS) was extracted from the high‐energy tail of the resulting absolute photoluminescence spectrum.

#### 7.3.7 Profilometry

Bruker Dektak XT profilometer was used to check the thickness of the perovskite thin films

#### 7.3.8 TRPL

Time‐resolved photoluminescence (TRPL) measurements were performed on a commercial fluorescence spectrometer (Edinburgh Instruments FLS920). For excitation, a 635 nm picosecond pulsed laser (Picoquant LDH‐series) operating at 50 kHz repetition rate and a power of 1.7 µW at the sample position was used. The laser was focused to a spot size of ∼0.3 mm diameter, resulting in an excitation density of ∼1.5·10^6^ photons cm^−2^.

## Author Contributions

**Uma Kousalya Dangudubiyyam**: investigation, writing – original draft, methodology, formal analysis, data curation, conceptualization, visualization, software. **Raphael Pesch**: writing – review and editing, methodology. **Ozan Karakaya**: visualization, methodology. **Ralf Niemann**: resources. **Henry Weber**: resources. **Fabian Fertig**: resources. **Theresa Kuechle**: writing – review and editing. **Johannes Sutter**: conceptualization, writing – review and editing, supervision. **Faranak Sadegh**: methodology, writing – review and editing. **Gerardo Hernandez Sosa**: supervision, writing – review and editing. **Nils W. Rosemann**: methodology, writing – review and editing. **Ulrich W. Paetzold**: conceptualization, writing – review and editing, supervision, funding acquisition. **Jinzhao Li**: conceptualization, writing – review and editing, supervision.

## Conflicts of Interest

The authors declare no conflicts of interest.

## Supporting information




**Supporting File**: advs76463‐sup‐0001‐SuppMat.docx.

## Data Availability

The data that support the findings of this study are available from the corresponding author upon reasonable request.
