# Supplementary material for: Eco‐Friendly Solvent System for Inkjet Deposition of Wide Bandgap Perovskite Solar Cells Enabling Tandem Integration
Source: Adv Sci (Weinh). 2026 Jul 9:e76463. Online ahead of print. doi: 10.1002/advs.76463 (PMC13348342; doi:10.1002/advs.76463)
Supplement: Supplementary file 1 — Supporting File: advs76463‐sup‐0001‐SuppMat.docx. [file ADVS-9999-e76463-s001.docx]

# Supplementary Information

**Eco-Friendly Solvent System for Inkjet Deposition of Wide Bandgap Perovskite Solar Cells Enabling Tandem Integration**

Uma Kousalya Dangudubiyyam * ^a, b^, Raphael Pesch ^a, c^, Ozan Karakaya ^a, b^, Theresa Kuechle ^a, b^, Faranak Sadegh ^a, c^, Nils W. Rosemann ^a^, Henry Weber ^e^, Ralf Niemann ^e^, Fabian Fertig ^e^, Johannes Sutter ^a, b, c^, Jinzhao Li * ^a, b, c^, Gerardo Hernandez Sosa ^a, b, c, d^, and Ulrich W. Paetzold * ^a, b, c^

1. *Light Technology Institute (LTI), Karlsruhe Institute of Technology, Engesserstrasse 13, 76131 Karlsruhe, Germany*
2. *InnovationLab, Speyerer Straße 4, 69115 Heidelberg, Germany*
3. *Institute of Microstructure Technology, Karlsruhe Institute of Technology,* *Hermann-von-Helmholtz-Platz 1, 76344 Eggenstein-Leopoldshafen, Germany*
4. *Institute for Automation and Applied Informatics, Karlsruhe Institute of Technology, Hermann-von-Helmholtz-Platz 1, 76344 Eggenstein-Leopoldshafen, Germany*
5. *Hanwha Q CELLS GmbH, Bitterfeld-Wolfen, Germany.*

[*uma.dangudubiyyam@kit.edu*](mailto:uma.dangudubiyyam@kit.edu) , [*ulrich.paetzold@kit.edu*](mailto:ulrich.paetzold@kit.edu)

Keywords:

Inkjet printing, Perovskite solar cell, Green solvents, Sustainable photovoltaics, γ-Valerolactone (GVL), Tandem solar cell

Table of Contents:

| Table S1 | 2 | Environmental Hazard Index analysis |
| --- | --- | --- |
| Table S2 | 2,3 | Safety Hazard Index analysis |
| Table S3 | 3 | Toxicity Hazard Index analysis |
| Table S4 | 4 | Health Hazard Index analysis |
| Figure S1 | 5 | Categorization of the greenness of a solvent |
| Figure S2 | 5 | Concentration-dependent solubility of perovskite ink |
| Figure S3 | 5 | Effect of silica nanoparticle concentration |
| Figure S4 | 6 | Optimization of vacuum quenching time |
| Figure S5 | 6 | XRD of perovskite films |
| Figure S6 | 7 | SEM of perovskite thin films with and without MACl |
| Figure S7 | 7 | TRPL of perovskite thin films with and without (PDAI_2_-BAI) |
| Figure S8 | 8 | Absolute PL on perovskite thin films with and without MACl |
| Figure S9 | 8 | *J-V* characterizations of perovskite solar cells |
| Table S5 | 9 | Photovoltaic parameters of inkjet-printed perovskite solar cells |
| Figure S10 | 9 | EQE of the perovskite top cell |
| Table S6 | 10 | Literature on inkjet printing |
| Figure S11 | 11 | Optical transmittance of perovskite thin film |
| Figure S12 | 11 | Repeatability of the developed PSC process |

Table S1: Environmental Hazard Index values for the polar aprotic solvents commonly used in perovskite research.

| *Solvent* | *Daphina Magna (EC50)* | *Bio-degradability* | *Reference* |
| --- | --- | --- | --- |
| DMAc | 500 mg/l | 28 days - 70% | [1] |
| DMF | 13.1 mg/ml | 21 days - 100% | [2] |
| NMP | 4.897 mg/ml | 28 days - 83% | [3] |
| DMI | 100 mg/ml | 28 days - 5% | [4] |
| Acetonitrile | 400mg/ml | 70% | [5] |
| THF | 3.485 mg/l | 28 days - 39% | [6] |
| GBL | 500 mg/l | 14 days - 95% | [7] |
| IPA | 13.299 mg/l | 5 days - 53% | [8] |
| GVL | 100 mg/ml | 28 days - 87% | [9] |
| Ethanol | 5.012 mg/ml | 15 days - 95% | [10] |
| DMSO | 24.6 mg/ml | 28 days - 31% | [11] |

Table S2: Safety Index values for the polar aprotic solvents commonly used in perovskite research.

| *Solvent* | *Flammability* | *Reactivity* | *Stability* | *Remarks* | *Reference* |
| --- | --- | --- | --- | --- | --- |
| DMAc | Moderate (Flashpoint 70°C) | Low | Stable | Can be absorbed through skin, with moderate toxicity; safer handling is required | [1] |
| DMF | Moderate (Flashpoint 58°C) | Low | Stable | Can be absorbed through skin, with moderate toxicity; safer handling is required | [2] |
| NMP | Low (Flashpoint 86°C) | Low | Stable | Low flammability, but toxic on prolonged exposure | [3] |
| DMI | Low (Non-flammable) | Low | Hygroscopic | Safe but absorbs moisture | [4] |
| Acetonitrile | High (Flashpoint 2°C) | Low | Stable | Flammable, toxic vapors | [5] |
| THF | High (Flashpoint -14°C) | Form explosive peroxides | Moderate | High flammability, peroxide formation risk | [6] |
| GBL | Low (Non-flammable) | Moderate (hydrolyzes to GHB) | Stable | Low physical hazard, but a potential drug precursor | [7] |
| IPA | High (Flashpoint 12°C) | Low | Stable | Flammable, but widely used safely | [8] |
| GVL | Low (Non-flammable) | Low | Stable | Considered a green solvent with minimal hazards | [9] |
| Ethanol | High (Flashpoint 13°C) | Low | Stable | Flammable, but widely used safely | [10] |
| DMSO | Non-flammable | Low | Stable | Safe handling, but enhances skin absorption | [11] |

Table S3: Toxicity Index values for the polar aprotic solvents commonly used in perovskite research.

| *Solvent* | *Oral LD50 (rat)* | *Reference* |
| --- | --- | --- |
| DMAc | 4300 mg/kg | [1] |
| DMF | 3010 mg/kg | [2] |
| NMP | 3914 mg/kg | [3] |
| DMI | 2000 mg/kg | [4] |
| Acetonitrile | 617 mg/kg | [5] |
| THF | 1650 mg/kg | [6] |
| GBL | 1540 mg/kg | [7] |
| IPA | 5045 mg/kg | [8] |
| GVL | 5000 mg/kg | [9] |
| Ethanol | 7060 mg/kg | [10] |
| DMSO | 14500 mg/kg | [11] |

Table S4: Health Hazard Index values for the polar aprotic solvents commonly used in perovskite research.

| *Solvent* | *Acute Toxicity (Oral, Dermal, Inhalation)* | *Carcinogenicity* | *Reproductive Toxicity* | *Other Hazards* | *Reference* |
| --- | --- | --- | --- | --- | --- |
| DMAc | Category 4 (oral, dermal, Inhalation) | Not classified | Category 1B (may impair fertility or cause harm to the unborn child) | Skin & eye irritation | [1] |
| DMF | Category 4 (oral, dermal, Inhalation) | Not classified | Category 1B (reprotoxic) | Liver toxicity, skin absorption | [2] |
| NMP | Category 4 (oral, dermal, Inhalation) | Not classified | Category 1B (reproductive toxicity - affects fertility and the unborn child) | Eye & skin irritation | [3] |
| DMI | Category 4 (oral, dermal) | Not classified | Category 1B (reprotoxic) | Skin irritation | [4] |
| Acetonitrile | Category 4 (oral, dermal, Inhalation) | Not classified | Not classified | Produces cyanide in metabolism | [5] |
| THF | Category 4 (oral, Inhalation) | Category 2 (suspected carcinogen - liver tumors in animals) | Not classified | Flammable, CNS effects | [6] |
| GBL | Category 4 (oral, dermal, Inhalation) | Not classified | Specific target organ toxicity - single exposure (Category 3) | CNS depressant | [7] |
| IPA | Category 4 (oral, Inhalation) | Not classified | Not classified | Irritation, CNS effects | [8] |
| GVL | Category 5 (low acute toxicity) | Not classified | Not classified | Skin Irritation | [9] |
| Ethanol | Category 5 (low acute toxicity) | Not classified | Not classified | CNS depressant | [10] |
| DMSO | Category 4 (oral, dermal) | Not classified | Not classified | Skin absorption enhancer | [11] |

****Figure S1: Categorization of the greenness of a solvent for various polar aprotic solvents commonly used in perovskite research.


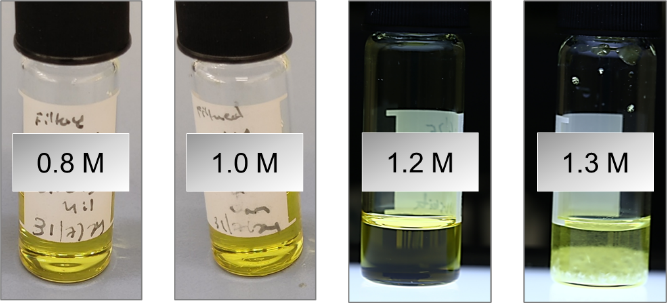


Figure S2: Concentration-dependent solubility of perovskite ink.


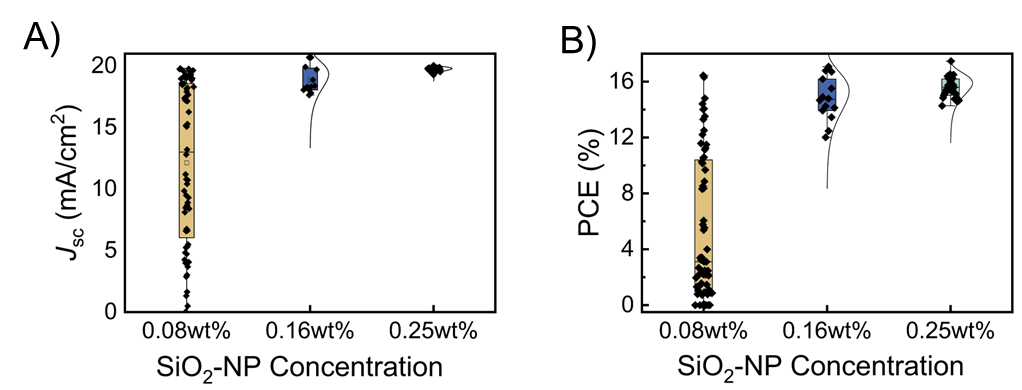


Figure S3: Effect of silica nanoparticle concentration on perovskite solar cell parameters. A) current density (*J*_SC_) and B) power conversion efficiency (PCE) of perovskite solar cells fabricated with 0.08, 0.16, and 0.25 wt% of SiO_2_-NP.


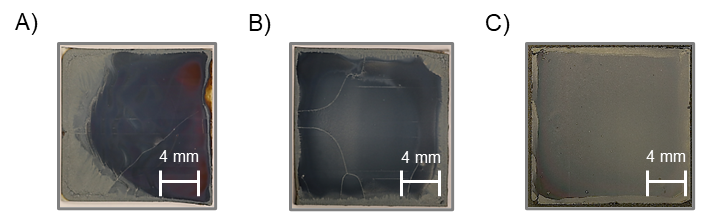


Figure S4: Optimization of vacuum quenching time. Drying patterns observed upon quenching for varying durations to achieve the intermediate δ-phase: A) quenching for 5 minutes, B) quenching for 10-30 seconds, and C) quenching for 60 seconds.


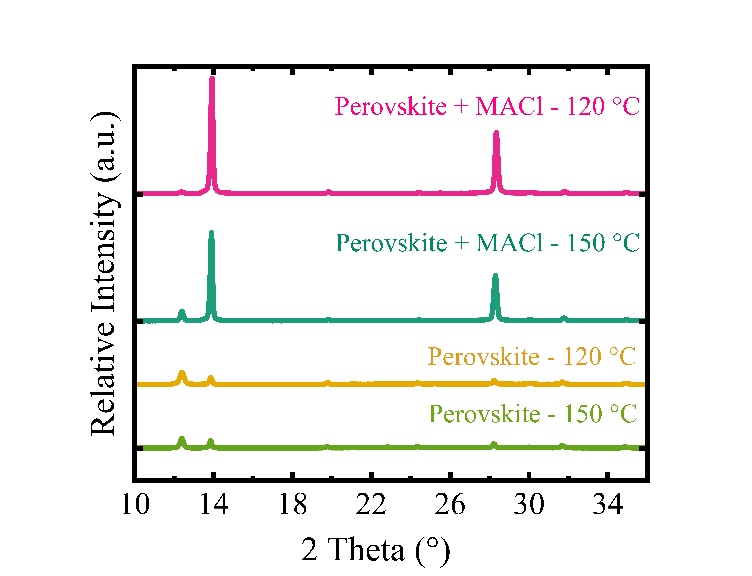


Figure S5: X-ray diffraction (XRD) patterns on perovskite thin film with and without MACl, fabricated at different temperatures. (ITO-coated glass/2PACz/SiO_2_-NP/Perovskite thin film).


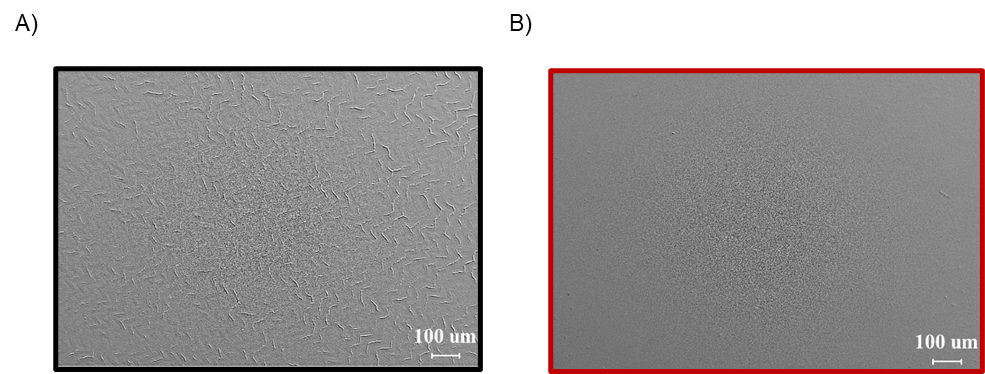


Figure S6: Scanning electron microscopy images of perovskite thin film surfaces, A) without MACl (outlined black) and B) with MACl (outlined red): The layer sequence of the measured samples is as follows: ITO-coated glass/2PACz/SiO_2_-NP/Perovskite thin film.


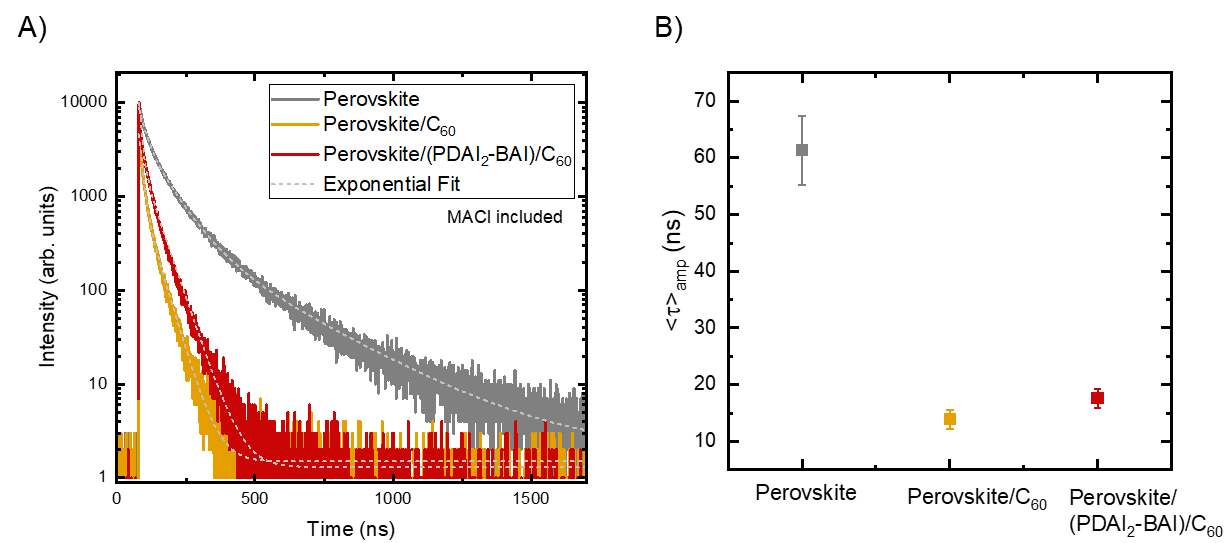


Figure S7: A) Time-resolved Photoluminescence (TRPL) transients and corresponding exponential fits for the bare perovskite (grey), perovskite/C_60_ (yellow), and perovskite/(PDAI_2_-BAI)/C_60_ (red) samples. A triple-exponential decay model was used for grey and red samples; for the yellow sample, a double-exponential fit was used, B) Amplitude-averaged lifetimes extracted from the exponential fits to the TRPL data.

Figure S8: Photoluminescence spectra of perovskite thin films. The layer sequence of the measured films is as follows: ITO-coated glass/2PACz/SiO_2_-NP/Perovskite thin film.


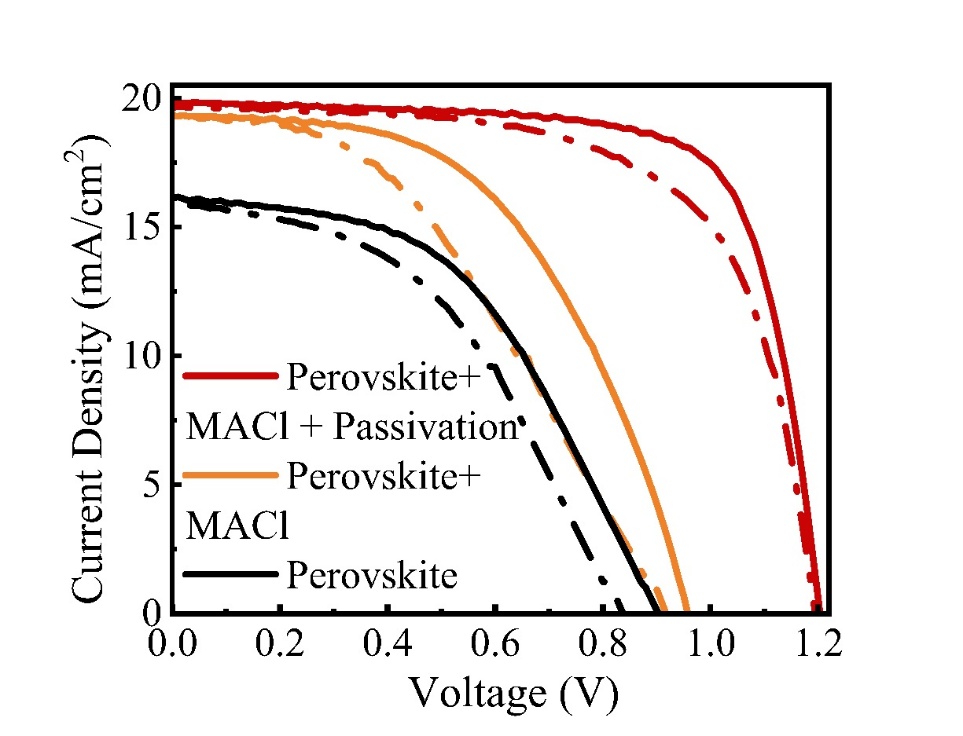


Figure S9: Effect of MACl and passivation (PDAI_2_-BAI) on *J-V* characterizations of perovskite solar cells.

Table S5: Photovoltaic parameters of the champion perovskite solar cells fabricated with green solvents and inkjet printing.

|  | *J*_SC_ (mA/cm^2^) | | *V*_OC_ (V) | | FF (%) | | PCE (%) | |
| --- | --- | --- | --- | --- | --- | --- | --- | --- |
| Perovskite | 16.1 | 15.9 | 0.93 | 0.83 | 48.8 | 45.4 | 7 | 6 |
| Perovskite + MACl | 19.3 | 19.3 | 0.95 | 0.91 | 52.3 | 41.4 | 9.6 | 7.3 |
| Perovskite + MACl + Passivation | 19.8 | 19.6 | 1.2 | 1.19 | 73.2 | 65.3 | 17.5 | 15.3 |

Figure S10: External quantum efficiency (EQE) and absorbance of perovskite top cell in tandem solar cell architecture.

Table S6: Literature published on perovskite solar cells fabricated using inkjet printing.

| *Solvent* | *Year* | *Deposition Technique* | *Perovskite Composition* | *PCE (%)* | *Reference* |
| --- | --- | --- | --- | --- | --- |
| DMF | 2014 | Inkjet printing | MAPbI_3_ | 11.6 | [12] |
| GBL | 2015 | Inkjet printing | MAPbI_3_ | 12.3 | [13] |
| GBL + DMSO | 2016 | Inkjet printing | MAPbI_3_ | 11.3 | [14] |
| 5-ammonium  valeric acid iodide + GBL | 2017 | Inkjet printing | MAPbI_3_ | 8.47 | [15] |
| DMSO + GBL | 2018 | Inkjet printing | MAPbI_3_ | 17.04 | [16] |
| DMF + DMSO + GBL | 2019 | Inkjet printing | Cs_0.10_FA_0.75_MA_0.15_Pb(Br_0.15_I_0.85_)_3_ | 21.6 | [17] |
| NMP + DMF + DMSO | 2020 | Inkjet printing | Cs_0.05_MA_0.14_FA_0.81_PbI_2.55_Br_0.45_ | 19.6 | [18] |
| NMP + DMF | 2021 | Inkjet printing | Cs_0.05_MA_0.14_FA_0.81_PbI_2.55_Br_0.45_ | 16.64 | [19] |
| GBL + 2MP + DMSO | 2021 | Inkjet printing | FAMACsPb(I_0.83_ Br_0.17_)_3_ | 11.4 | [20] |
| DMF | 2022 | Inkjet printing | MAPbI*_x_*Cl_3–_*_x_* | 10.85 | [21] |
| GVL | 2023 | Inkjet printing | MAPbI_3_ | 13.07 | [22] |
| DMF + DMSO + Acetonitrile | 2025 | Inkjet printing | FAMAPb(Br)_3_ | 13.44 | [23] |
| GVL + (DMF + DMSO + DMI) | 2025 | Inkjet printing | Cs_0.10_FA_0.75_MA_0.15_Pb(Br_0.15_I_0.85_)_3_ | 17.78 | [24] |
| GVL + DMSO | 2025 | Inkjet printing | FAMACsPb(I_0.77_ Br_0.23_)_3_ | 17.5 | This Work |
| GVL + DMSO | 2025 | Inkjet printing | Silicon bottom cell + FAMACsPb(I_0.77_ Br_0.23_)_3_ | 28.1 | This Work |

The optical transmittance spectrum of the perovskite thin film deposited on glass(1.1 mm)/ITO/2PACz/SiO_2_-NP is provided in Figure S11, Supplementary Information

Figure S11: Optical transmittance of perovskite thin film on (Glass/ITO/2PACz/SiO_2_-NP).


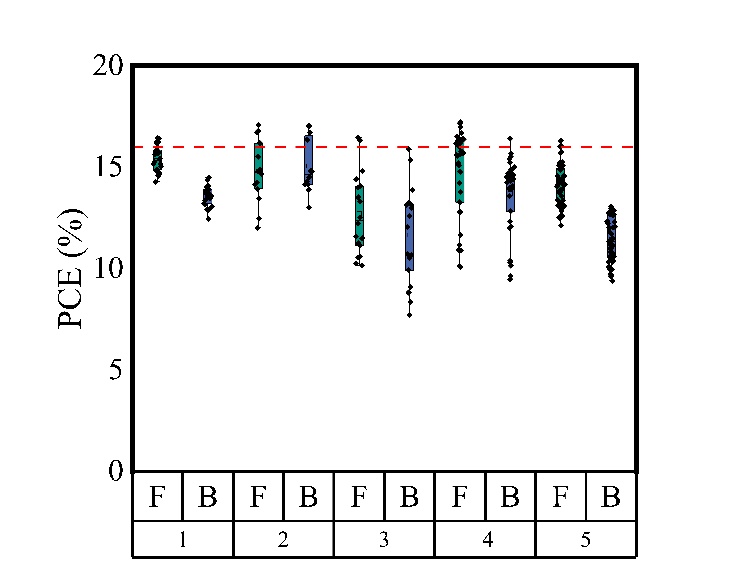
The reproducibility of the device performance across various batches, where perovskite is inkjet-printed in ambient conditions, is shown in Figure S12, Supplementary Information.

Figure S12: Repeatability of the developed perovskite solar cell process (F: Forward, B: Backward, and the numbers indicate the batch count).

## References:

[1] Sigma-Aldrich, “Dimethylacetamide Safety Data Sheet.” Accessed: Jul. 07, 2025. [Online]. Available: https://www.sigmaaldrich.com/DE/en/sds/sial/271012

[2] Sigma-Aldrich, “Dimethylformamide Safety Data Sheet.” Accessed: Jul. 07, 2025. [Online]. Available: https://www.sigmaaldrich.com/DE/en/sds/SIAL/227056

[3] Sigma-Aldrich, “N-Methyl-2-pyrrolidone (NMP) Safety Data Sheet.” Accessed: Jul. 07, 2025. [Online]. Available: https://www.sigmaaldrich.com/DE/en/sds/SIGALD/270458

[4] Sigma-Aldrich., “1,3-Dimethyl-2-imidazolidinone (DMI) Safety Data Sheet.” Accessed: Jul. 07, 2025. [Online]. Available: https://www.sigmaaldrich.com/DE/en/sds/aldrich/40727

[5] Sigma-Aldrich., “Acetonitrile Safety Data Sheet.” Accessed: Jul. 07, 2025. [Online]. Available: https://www.sigmaaldrich.com/DE/en/sds/sial/271004

[6] Sigma-Aldrich, “Tetrahydrofuran (THF) Safety Data Sheet.” Accessed: Jul. 07, 2025. [Online]. Available: https://www.sigmaaldrich.com/DE/en/sds/sial/401757

[7] Sigma-Aldrich, “Gamma-Butyrolactone (GBL) Safety Data Sheet.” Accessed: Jul. 07, 2025. [Online]. Available: https://www.sigmaaldrich.com/DE/en/sds/aldrich/b103608

[8] Sigma-Aldrich, “Isopropyl alcohol (IPA) Safety Data Sheet.” Accessed: Jul. 07, 2025. [Online]. Available: https://www.sigmaaldrich.com/DE/en/sds/sial/w292912

[9] Sigma-Aldrich, “Gamma-Valerolactone (GVL) Safety Data Sheet.” Accessed: Jul. 07, 2025. [Online]. Available: https://www.sigmaaldrich.com/DE/en/sds/aldrich/w310301

[10] Sigma-Aldrich, “Ethanol Safety Data Sheet.” Accessed: Jul. 07, 2025. [Online]. Available: https://www.sigmaaldrich.com/DE/en/sds/sial/459836

[11] Sigma-Aldrich, “Dimethyl sulfoxide (DMSO) Safety Data Sheet.” Accessed: Jul. 07, 2025. [Online]. Available: https://www.sigmaaldrich.com/DE/en/sds/SIGMA/D2438

[12] Z. Wei, H. Chen, K. Yan, and S. Yang, “Inkjet Printing and Instant Chemical Transformation of a CH3NH3PbI3/Nanocarbon Electrode and Interface for Planar Perovskite Solar Cells,” *Angewandte Chemie*, vol. 126, Jul. 2014, doi: 10.1002/ange.201408638.

[13] S. G. Li *et al.*, “Inkjet printing of CH3NH3PbI3 on a mesoscopic TiO2 film for highly efficient perovskite solar cells,” *J Mater Chem A Mater*, vol. 3, no. 17, pp. 9092–9097, May 2015, doi: 10.1039/c4ta05675b.

[14] F. Mathies *et al.*, “Multipass inkjet printed planar methylammonium lead iodide perovskite solar cells,” *J Mater Chem A Mater*, vol. 4, no. 48, pp. 19207–19213, 2016, doi: 10.1039/c6ta07972e.

[15] S. G. Hashmi *et al.*, “Air Processed Inkjet Infiltrated Carbon Based Printed Perovskite Solar Cells with High Stability and Reproducibility,” *Adv Mater Technol*, vol. 2, no. 1, Jan. 2017, doi: 10.1002/admt.201600183.

[16] C. Liang *et al.*, “One-Step Inkjet Printed Perovskite in Air for Efficient Light Harvesting,” *Solar RRL*, vol. 2, no. 2, Feb. 2018, doi: 10.1002/solr.201700217.

[17] H. Eggers *et al.*, “Inkjet-Printed Micrometer-Thick Perovskite Solar Cells with Large Columnar Grains,” *Adv Energy Mater*, vol. 10, no. 6, p. 1903184, 2020, doi: https://doi.org/10.1002/aenm.201903184.

[18] Z. Li *et al.*, “Ink Engineering of Inkjet Printing Perovskite,” *ACS Appl Mater Interfaces*, vol. 12, no. 35, pp. 39082–39091, Sep. 2020, doi: 10.1021/acsami.0c09485.

[19] L. Zhang *et al.*, “Ambient Inkjet-Printed High-Efficiency Perovskite Solar Cells: Manipulating the Spreading and Crystallization Behaviors of Picoliter Perovskite Droplets,” *Solar RRL*, vol. 5, no. 5, May 2021, doi: 10.1002/solr.202100106.

[20] B. Wilk *et al.*, “Green Solvent-Based Perovskite Precursor Development for Ink-Jet Printed Flexible Solar Cells,” *ACS Sustain Chem Eng*, vol. 9, no. 10, pp. 3920–3930, Mar. 2021, doi: 10.1021/acssuschemeng.0c09208.

[21] D. A. Chalkias, A. Mourtzikou, G. Katsagounos, A. Karavioti, A. N. Kalarakis, and E. Stathatos, “Suppression of Coffee-Ring Effect in Air-Processed Inkjet-Printed Perovskite Layer toward the Fabrication of Efficient Large-Sized All-Printed Photovoltaics: A Perovskite Precursor Ink Concentration Regulation Strategy,” *Solar RRL*, vol. 6, no. 8, Aug. 2022, doi: 10.1002/solr.202200196.

[22] D. A. Chalkias, A. Mourtzikou, G. Katsagounos, A. N. Kalarakis, and E. Stathatos, “Development of Greener and Stable Inkjet-Printable Perovskite Precursor Inks for All-Printed Annealing-Free Perovskite Solar Mini-Modules Manufacturing,” *Small Methods*, vol. 7, no. 10, p. 2300664, 2023, doi: https://doi.org/10.1002/smtd.202300664.

[23] D. Lu, M. Jamshidi, J. M. Gardner, and L. Belova, “Scalable Fabrication of Perovskite Solar Cells with Inkjet-Printed Perovskite Absorbers Processed under Ambient Conditions,” *ACS Appl Mater Interfaces*, vol. 17, no. 19, pp. 28055–28064, May 2025, doi: 10.1021/acsami.4c20567.

[24] V. V. Satale *et al.*, “Green Solvent Enabled Perovskite Ink for Ambient-Air-Processed Efficient Inkjet-Printed Perovskite Solar Cells,” *Adv Funct Mater*, Oct. 2025, doi: 10.1002/adfm.202503717.
